# Findings of a community screening programme for human cystic echinococcosis in a non-endemic area

**DOI:** 10.1371/journal.pgph.0000235

**Published:** 2022-08-15

**Authors:** Titus Mutwiri, Japhet Magambo, Eberhard Zeyhle, Anne W. T. Muigai, Lorren Alumasa, Fredrick Amanya, Eric M. Fèvre, Laura C. Falzon

**Affiliations:** 1 Jomo Kenyatta University of Agriculture and Technology, Nairobi, Kenya; 2 International Livestock Research Institute, Nairobi, Kenya; 3 Kenya Methodist University, Nairobi, Kenya; 4 Meru University of Science and Technology, Meru, Kenya; 5 Institute of Infection, Veterinary and Ecological Sciences, University of Liverpool, Liverpool, United Kingdom; Institute of Development Studies, UNITED KINGDOM

## Abstract

Cystic Echinococcosis (CE) is a zoonosis caused by infection with the larval stages of the taeniid cestodes of the species complex *Echinococcus granulosus* sensu lato. It is prevalent among transhumant communities in East Africa, including those residing in northern Kenya. The movement of livestock from these regions of high incidence to areas of low incidence creates an indirect risk of disease spill-over to humans. To assess possible establishment of the CE life cycle outside known endemic regions, we used a portable ultrasound scanner to screen for the presence of human CE in Bungoma County of western Kenya, an area which imports substantial numbers of cattle for slaughter from neighbouring pastoralist regions. Eight sentinel sites were purposively selected based on their proximity to slaughterhouses handling animals introduced from pastoralist regions, and necessary permissions to conduct the study were sought. Regression analyses were conducted to identify risk factors associated with the presence of abdominal and cystic lesions (CL). In total, 1002 participants were screened; of these, 654 (65.3%) were female and the median age was 43. Farming (n = 403; 43.4%) was the most frequent occupation, followed by professional (i.e. on regular salary) (n = 215; 23.1%), and business (n = 207; 22.3%) categories. Sixty-seven participants (6.7%) had abnormal ultrasound findings, of these, 7 (1.1%) had simple liver cysts/CL, as per WHO classification. As such, their outcome was inconclusive and they were not put on treatment but advised to attend follow-up investigations in a referral health facility. Other abnormal findings included splenomegaly (n = 14), ovarian cysts (n = 14), uterine fibroids (n = 10), polycystic kidneys (n = 6), and benign prostatic hyperplasia (n = 6). Age was unconditionally associated with the presence of presumptive CL. These results contribute to CE baseline data while providing insights on the implementation of ultrasound diagnosis in the field, as recommended by the WHO for targeted control of echinococcosis by 2030.

## Introduction

Cystic Echinococcosis (CE) is a zoonotic disease caused by the tapeworm *Echinococcus granulosus* sensu lato (s.l.) and is of worldwide public health importance [1]. The disease causes considerable economic losses and public health problems in many countries [2, 3], and more significantly where livestock farming is heavily practised. Species that cause human CE include *E*. *granulosus* sensu stricto *(s*.*s)* (genotypes G1 and G3), *E*. *canadensis* (G6, G7, G8 and G10), *E*. *ortleppi* (G5), and *E*. *equinus* (G4) [4].

The disease is mainly transmitted by canids and has an array of intermediate hosts, primarily ungulates such as sheep, goat, buffalo, horses, cattle, pigs, camels, and cervids [5]. In the typical life cycle, tapeworm eggs are passed in the faeces of an infected dog and may subsequently be ingested by grazing sheep [6, 7]. The eggs then hatch into oncospheres in the intestine, penetrate actively through the intestinal wall, and are carried passively by blood or lymph fluid to organs–primarily the liver and lungs–where they settle and transform into tiny Echinoccocus cysts. These cysts grow with time and produce asexually into protoscolices (small tapeworms). The lifecycle is completed when final canid hosts are either fed offal containing Echinococcus cysts, or access them through scavenging or following home slaughter of ruminants [8]. Humans can serve as aberrant hosts when they become infected after ingesting tapeworm eggs excreted by infected canids. This frequently occurs when individuals handle or come into contact with infected dogs or other infected carnivores, or inadvertently ingest food or drink water contaminated with faecal material containing tapeworm eggs [6]. The hydatid cysts in humans tend to grow slowly, but they can have a prolonged lifespan of over 50 years within the infected organs [9], where the slow but continuous growth may cause obstruction and mechanical pressure leading to pathological compression.

In Kenya, CE has long been prevalent among the pastoralist and transhumant communities [10–12]. In the early 1950s, CE was reported to primarily affect a focus in the northern, arid Turkana County, with particularly high infection levels in the north-west and north-east regions; later, the Pokot and Maasai communities also reported a small incidence of human CE [13]. We consider the opening up and growth of the animal trade [14] involving movement of livestock from regions of high disease incidence to areas of low incidence, to be a risk for the spread of diseases among animals in otherwise low risk regions in Kenya, with the consequent hazard of spill-over to humans. Indeed, Kenya is continuously recording CE infections in livestock and dogs, as evidenced in studies in Masailand [15], Turkana, Maasai Mara, Isiolo and Meru [16], as well as central regions of Kenya and their neighbourhood [17], highlighting the growing need to assess possible spill-over of infections to human populations. This, and other similar assessments, would contribute information on the distribution and pathogenicity of prevailing *Echinococcus* taxa in these regions, allowing for targeted and locally adapted prevention and control efforts [18], which would support the World Health Organization (WHO) 2030 Neglected Tropical Diseases (NTD) action plan [19].

Ultrasound (US) screening is considered the gold standard for CE diagnosis in humans as it is non-invasive and painless, making it widely accepted by patients [20]. Moreover, US screening allows for the classification of hydatid cysts, which in turn may inform the treatment options. These include: “Watch-and-Wait” for uncomplicated inactive cysts, or surgery, percutaneous treatments and use of chemotherapeutic agents for viable cysts [21]. Besides cyst characteristics, the choice of the optimal treatment option is also guided by available medical and surgical expertise, health care facilities, and the patient’s willingness to participate in long-term monitoring [22]. Ultrasound screening also allows for the diagnosis of other clinically important lesions in the liver, hepatobiliary–pancreatic system, and urogenital tracts. Although the use of ultrasound may further exacerbate the economic burden of the participants through discovery of incidental findings, the overriding advantage is in its ability to guide therapeutic decisions even in remote and medically underserviced areas that lack diagnostic facilities, while also contributing to knowledge on the burden of such conditions in these areas.

Since the description of *Echinococcus granulosus* in Kenya in the mid-20^th^ Century [10, 23–25], few human screening initiatives have been conducted, and these were primarily among the Turkana and the Maasai communities [13]. Therefore, the primary objective of this study was to screen for human CE in Bungoma county, western Kenya, to help assess the possible establishment of the CE life cycle outside the endemic region, linked to the cattle trade. Moreover, the secondary outcome was to assess for the presence of other abdominal lesions within the same population. In extension the outcome of this study would help guide long-term decisions for control and prevention of intra-country transmission of CE. The western Kenya region represents the larger Lake Victoria Basin ecosystem, a region with the highest rural human and livestock population densities in Eastern Africa, and where the mixed smallholder livestock production system predominates [26]. Furthermore, Bungoma County serves as an entry route of livestock from areas of high CE prevalence, such as Turkana and Pokot, into western Kenya, with consequent impacts on disease transmission.

## Materials and methods

### Study area

**Fig 1:**
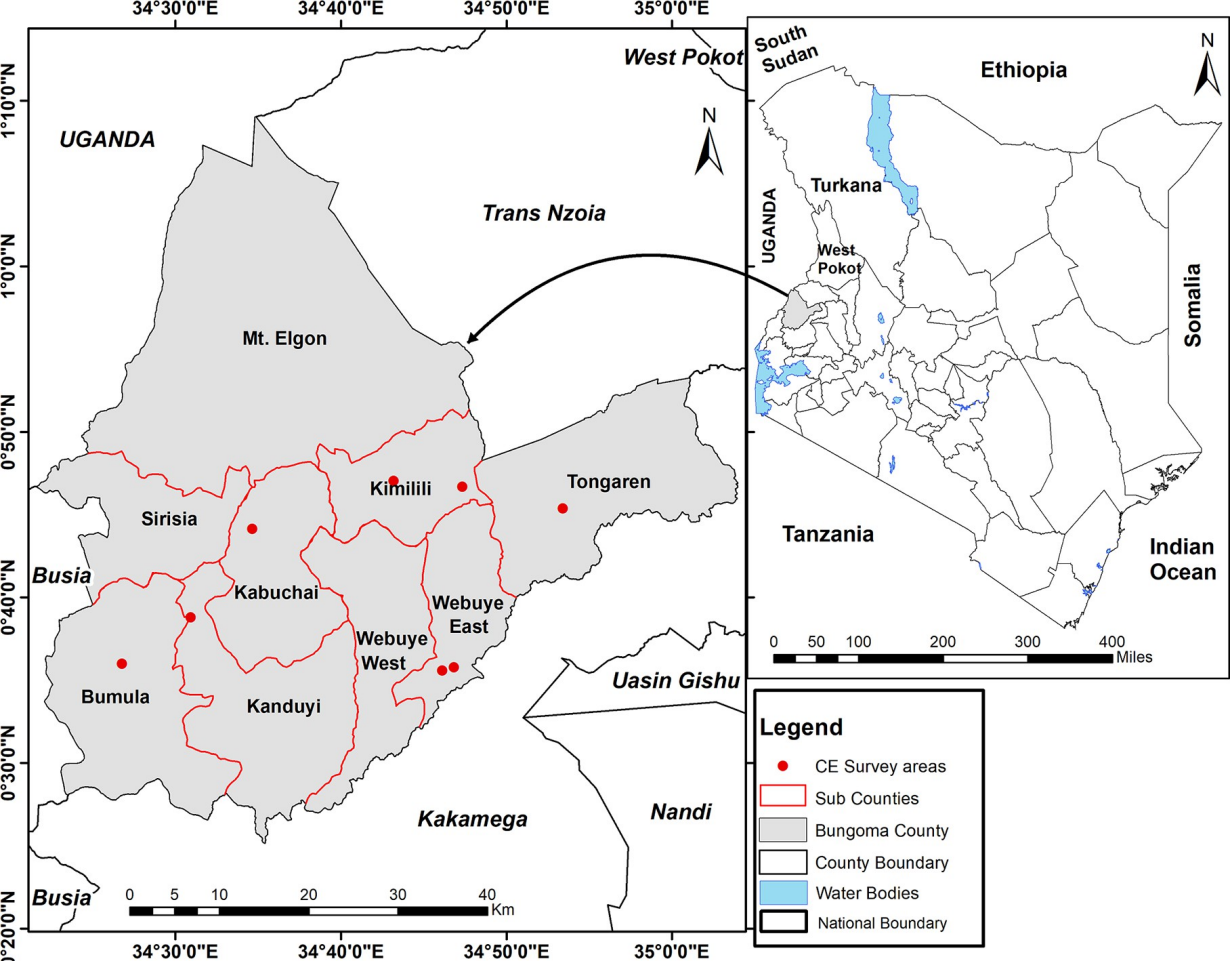
A map of Kenya showing the location of the sentinel sites within Bungoma County. The base layer datasets included vectors files (shapefiles) of Kenya subnational boundaries. That is, county shapefiles sourced from Landsat [28], subcounty shapefiles were sourced from Figshare [29]. The water shapefiles were sourced from The Landsat [28]. The CE surveys data points were collected as part of the study as described in the manuscript. The map was created using ArcMap version 10.5 (ESRI Inc., Redlands, CA, USA).

Bungoma County sits within the former western province of Kenya. The county has an area of 2,069 km^2^ and a population of 1,670,570, of which 812,146 are males and 858,389 females, as per the 2019 Kenya National Population census [27]. Only 12.5% of this population live in the urban or peri-urban areas, with the rest living in the rural parts of the county. The study was conducted in eight peri-urban sites in Bungoma County with a combined approximate population of 208,685 persons. The sites were: Naitiri, Misikhu, Wanaichi, Kimilili, Kamukuywa, Chwele, Mayanja, and Kimwanga (Fig 1).

### Study sites

The eight sentinel sites were purposively selected based on their proximity to livestock markets [30] where it is known that animals arrive from Turkana and Pokot and are sold for slaughter. Since animal products are a main source of food in western Kenya, while animal husbandry in the region is still predominately practised at a low scale, supply of livestock to meet food demand relies on the continued import of livestock from outside the immediate region, including from Turkana and west Pokot.

At each site, an area deemed appropriate and convenient for mass screening was identified, based on its proximity to the local community and points of convergence for other social activities. The points data for each site were recorded. The sites included: five hospitals (in Naitiri, Kamukuywa, Chwele, Mayanja, and Kimwanga), two chief’s camps (in Misikhu and Wanaichi), and one open market (in Kimilili). Hospitals were chosen for mass screening since they would serve not only as suitable sites of community convergence but also provide a conducive environment for screening (e.g., private rooms, electricity, etc.).

### Study design

Prior to the start of the study, each site was visited to inform the local administration, including chiefs and assistant chiefs, about the study plans, and to seek their permission to conduct the study. Subsequently, village elders were notified, who in turn assisted in sensitization of locals about the study. Additionally, the chiefs announced the screening dates during local *Barazas* (social administrative gatherings) and religious congregations held at each site.

Each site was visited once, on the scheduled day, by a research team that comprised an experienced sonographer, a clinical officer, a microbiologist, and two other research assistants. On each day, the research team aimed to reach the study site by 7 a.m. and remained on site until all who were willing to participate were screened. Posters with illustrative images of the parasite’s life cycle and its detrimental effects to humans and livestock were used to create awareness as well as to demonstrate the ultrasonography protocol. The visual aids also highlighted the risks of contracting the infection through contact with dog faeces, and the consequences of infection in human and livestock.

The study rationale was explained to any attendee who showed willingness to participate in the study. The participants were also informed that the US screening could detect other significant abdominal lesions and, if the participant consented and where applicable, images would be printed and explained to them, as per the WHO guidelines [31]. Plans were put in place for any eventual CE patients or emergent incidental finding requiring urgent referral be sent to a county referral hospital for further treatment and/or follow-up.

After the awareness session, willing participants signed consent forms; parents or guardians signed assent forms on behalf of minor participants. Prior to screening, participants responded to a brief questionnaire asking about their gender, age, place of origin, income level, and occupation. Any case of previous surgery, and the reason thereof, was also recorded. There was no pre-selection of participants based on clinical signs and as such no exclusion criteria was used; all those willing to participate were included in the study.

At each site, a cubicle or room enclosed with curtains was set up, where the sonographer and clinician could screen each participant in privacy using a portable US scanner. The scanner was electrical and generator-powered to ensure uninterrupted screening in case of power outages. The US findings were communicated to the participants and, when need arose, counselling by the clinical officer and hospital referral was done.

#### Ethics approval and consent to participate

This study was approved by the Institutional Research Ethics Committee (IREC) Reference No. 2018–02, at the International Livestock Research Institute (ILRI). The ILRI IREC is accredited by the National Commission for Science, Technology and Innovation (NACOSTI) in Kenya, and approved by the Federalwide Assurance (FWA) for the Protection of Human Subjects in the United States of America. Approval to conduct this work was also obtained from the Kenyan Ministry of Health and the relevant offices at devolved government level, and sub-county medical and public health officers.

### Interpretation of ultrasound images

**Fig 2:**
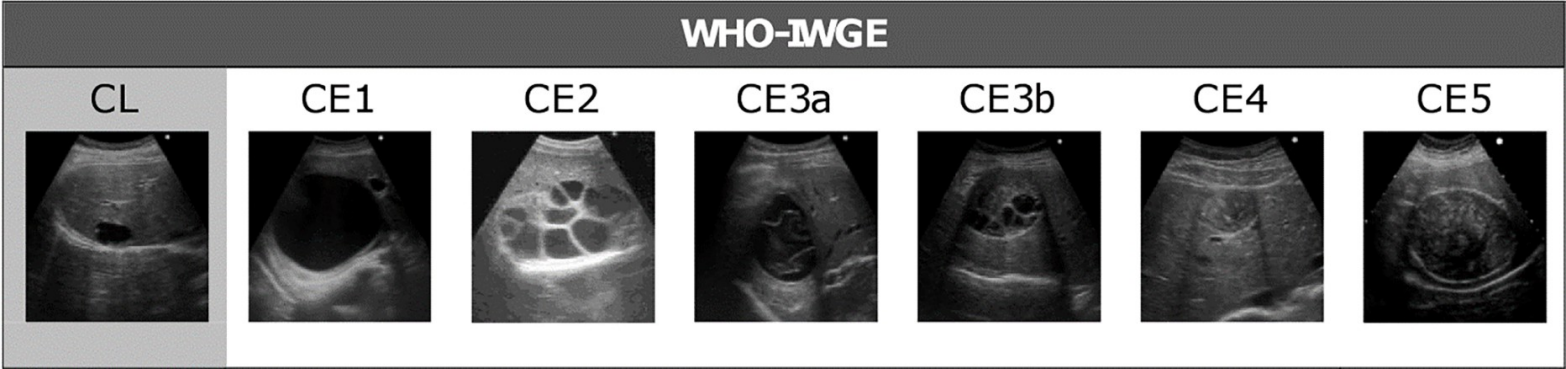
WHO Informal Working Group on Echinococcosis (IWGE) standardized ultrasound classification of echinococcal cysts. CE1 and CE2 (active cysts), CE3a and CE3b (transitional cysts), and CE4 and CE5 (inactive cysts) [32].

All US images were anonymised before further analysis. These images were screened for the presence of hydatid cysts or other abdominal lesions. Where applicable, the WHO staging classification for hepatic cyst(s), namely CL or CE1 to CE5 [31] was used. CE1 cysts present with a double wall; CE2 cysts reveal daughter cysts; CE3a show detached parasitic membranes; CE3b cysts present an image of daughter cysts in a partially solid matrix including anechoic folded parasite membranes (“ball-of-wool” sign); CE4 cysts are completely filled by a solid matrix including anechoic folded parasite membranes; whereas a CE4 cyst with an egg-shell-like calcification denotes a CE5 stage (Fig 2). The CL present as unilocular, cystic lesion(s) with uniform anechoic content, not clearly delimited by a hyperechoic rim (= cyst wall not visible). Cystic lesions may be round to oval and vary in size from <5.0 cm to >10 cm and should not be associated with cystic echinococcosis. However, if the cystic lesions are due to CE, then these cysts are usually at an early stage of development and are not fertile. Ultrasound screening is therefore unable to detect any pathognomonic signs in the case of CL cysts, and differential diagnosis requires further diagnostic techniques [22].

### Statistical analysis

Questionnaire data and US findings were first recorded on paper-based forms at the site, and later entered manually into Microsoft Excel (Microsoft, Redmond, WA, USA). Data cleaning was then carried out to check for any errors that might have occurred during transcription.

Statistical analysis was conducted using Stata Statistical Software: Release 14 (College Station, TX: StataCorp LP). Descriptive statistics were performed to summarize variables and identify trends. The entries provided for the explanatory variable “occupation” were categorized as: farmer, business (this included: tailors, motorcycle transport operators, and shopkeepers), professional (i.e. those on a regular salary e.g., nurses, teachers, and policemen), homestay (e.g. housewives or househusbands), and minor. A new explanatory variable–“occupation risk”–was created based on the occupation described by the participants, whereby farmer and homestay categories were considered as high risk, while business, professional, and minor categories were considered as low risk for human CE.

**Fig 3:**
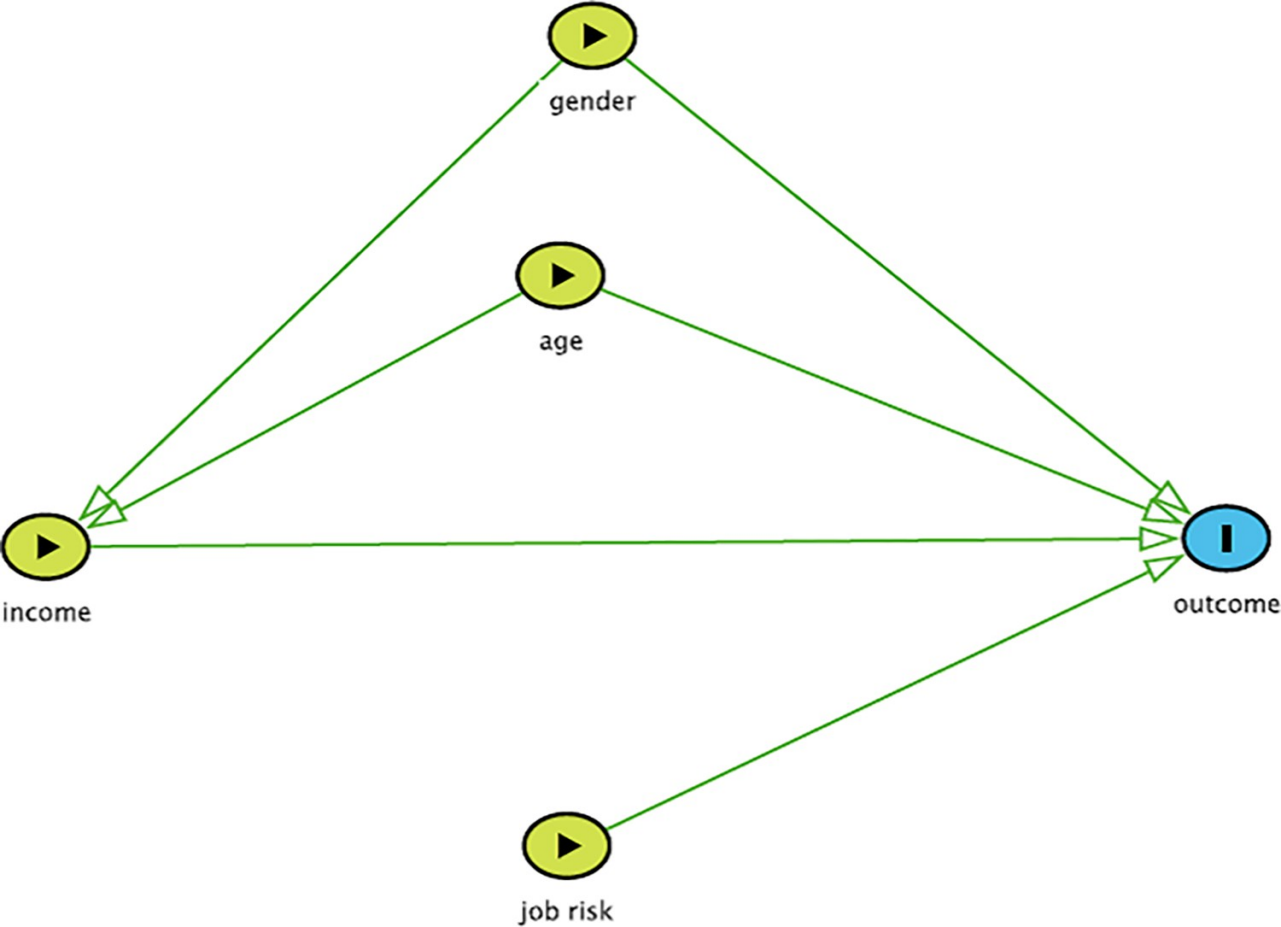
A causal diagram illustrating the putative relationships between the explanatory variables and the two outcomes of interest (presence of abnormal ultrasound findings and presence of liver cysts).

Regression analysis for the two outcomes of interest, namely abnormal US findings (Y/N) and presence of liver cysts (Y/N), were conducted. A causal diagram was developed to identify putative relationships between exposure variables of interest and outcomes, and to guide the modelling process (Fig 3). Continuous explanatory variables were checked for normal distribution; variables that were not normally distributed were transformed as needed. Subsequently, mixed logistic regression models were developed for each outcome variable, with site included as a random effect to account for spatial clustering within each sentinel site. Each explanatory variable was first screened for its unconditional association with the outcome variable; variables that were marginally significant (p<0.2) or considered confounding were retained for inclusion in the multivariable regression model. The intra-cluster coefficient was computed as the proportion of overall variation due to variation between groups.

## Results

The US screening took place over eight days, one day for each site. In total, 1002 participants were screened. Some study sites, such as Kimilili, had a larger turn-out since they are larger towns with bigger populations (Table 1). Almost twice (1.88 times) as many women, compared to men, participated in the study, and this was consistent across the sites, with the exception of Kimilili. The overall median age of the participants was 43 years (IQR = 29–55); the median age was slightly lower in females (43) than in males (49). The median monthly income was 4000 KES (US$ 36.50), though this was lower for females (3100 KES; US$ 28.29) compared to males (4200; US$ 38.32).

**Table 1:** Ultrasound screening across the sub-counties of Bungoma County.

Ultrasound results	Selected sub-counties of Bungoma								
	Chwele	Kamukuywa	Kimilili	Kimwanga	Mayanja	Misikhu	Naitiri	Wanaichi	Total
**Participants (M:F)**	83 (21:62)	102 (33:69)	218 (111:107)	155 (45:110)	124 (28:96)	75 (22: 53)	123 (45:78)	122 (43:79)	**1002 (438:654)**
**normal**	76	93	209	139	115	73	119	111	**935**
**abnormal**	7	9	9	16	9	2	4	11	**67**

Occupation data were available for 930 participants. Farming was the most frequent occupation category (n = 403; 43.4%), followed by professional (e.g. teachers, nurses and policemen) (n = 215; 23.1%), business (e.g. tailors, motorcycle transport operators, shopkeepers) (n = 207; 22.3%); minor (n = 78; 8.4%); and homestay (housewives and househusbands) (n = 27; 2.9%) categories. Consequently, 430 (46.2%) and 500 (53.8%) of the participants were classified as being at high and low risk for human CE, respectively.

Previous surgeries were reported by 100 participants; 40, however, did not disclose the type of surgery. Of the remaining 60 participants who reported a previous surgery, the most frequent was caesarean section (n = 29), followed by tubal ligation (n = 8), and growth removal (n = 3). Two participants reported previous removal of cysts, though details on the nature of the cysts were not provided.

Of the 1002 screened participants, 67 (6.7%) had abnormal US findings. Seven participants (0.7% of total, 10.4% of those with abnormal findings)—five females and two males—had simple liver cysts (ranging from 1.76cm x 2.92cm to 8.60cm x 8.02cm) which were classified as CL following the WHO classification. As such, no US findings could confirm the presence of CE in the study population.

Other abnormal US findings included mild to moderate splenomegaly (n = 14), ovarian cysts (n = 14), uterine fibroids (n = 10), polycystic kidneys (n = 6), and benign prostatic hyperplasia (n = 6). Abdominal ascites, cardiac insufficiency, cystic lesions in the left scapular and lower abdomen, echogenic lesions in the left upper arm, and gall stones were each identified once. A rare case of umbilicus lymphoma (Sister Mary Joseph’s nodule) was identified in a 40-year-old female, and two cases of hepatocellular carcinoma were identified. A possible ovarian tumour and uterine mass were also detected. These participants were all referred to an oncologist for further medical attention.

### Regression analysis

Of the four explanatory variables identified in the causal diagram (Fig 3), age and income were continuous and therefore assessed for normality; while age was normally distributed, income had a right-skewed distribution and was therefore log-transformed.

The results of the unconditional associations between the two outcome variables and the explanatory variables are presented in Table 2, while the results of the final mixed logistic regression model for abnormal US findings are presented in Table 3. The variables gender and age were retained in the final model, regardless of their p-values, since they were identified as confounders (Fig 3). No variables remained significant in the multivariable regression model for simple liver cysts, so no final model is presented.

**Table 2:** Unconditional associations between the putative risk factors and the two outcome variables—abnormal ultrasound findings and presence of liver cysts—from 1002 participants screened in eight sentinel sites in Bungoma County.

Explanatory variable	Outcome: Abnormal ultrasound findings			Outcome: Simple liver cysts		
	Odds Ratio	95% CI	p-value	Odds Ratio	95% CI	p-value
**Gender: Male [referent]**	0.64	0.36–1.13	0.128*	0.75	0.14–3.89	0.73
**Age**	1.01	0.99–1.03	0.085*	1.09	1.04–1.15	<0.001***
**Income_ln**	0.65	0.49–0.86	0.003**	2.32	0.77–6.96	0.133*
**Job risk: Low risk [referent]**	0.57	0.33–0.98	0.043**	0.86	0.17–4.28	0.85

*p<0.20

**p<0.05

***p<0.001

**Table 3:** Final mixed logistic regression model for abnormal ultrasound findings identified in 1002 participants screened in eight sentinel sites in Bungoma County.

	Odds Ratio	95% CI	p-value	ICC
**Site**				1.67^−9^
**Gender: Male [referent]**	0.45	0.22–1.03	0.060*	
**Age**	1.01	0.99–1.04	0.241	
**Income_ln**	0.68	0.51–0.90	0.007**	

ICC = Intra-Cluster Correlation Coefficient

## Discussion

In this study we conducted community US screening for the presence of CE and other abdominal lesions. These results contribute baseline data on field investigations for human CE while providing insights on the implementation of ultrasound diagnosis in the field, as recommended by the WHO for the successful targeted control of echinococcosis by 2030 [19].

Most participants who underwent screening were between 29 and 55 years old. While some parents/guardians brought their young children and assented for their screening, the population of pupils in school largely missed out since the study was done during the school season. Additionally, the 29–55 years age group were more receptive and keen to participate in the screening, compared to the younger or more elderly age groups.

Many of the participants were farmers whose livelihood depends on subsistence farming. Indeed, their income was lower (median income of KES 3000 / US$ 27.10 per month), compared to that of the business and professional categories (KES 5000 / US$ 45.10 and 6250 / US$ 56.38 per month, respectively). The study observed that the levels of earning by the participants would not be sufficient to cater for their basic needs and routine medical check-ups, thus explaining their willingness to participate in the free US screening programme.

Cystic echinococcosis infections in patients were identified using ultrasound on the basis of pathognomonic signs by clinicians skilled in ultrasonography and radiology. The CE cysts were staged according to the WHO-IWGE classification [22]. Pathognomonic signs of interest present in various forms and classification, and in this study, the seven cases of cystic lesions detected by the ultrasonogram presented as unilocular fluid-filled cysts with a visible single wall and were classified as cystic lesions of uncertain aetiology (referred to as CL in the WHO-IWGE classification). It was therefore not possible to form a definitive diagnosis as CL indicates undifferentiated cystic lesion that requires further investigation before definitive decision of its parasitic nature is made. However, when a CL is detected on a scan in countries or regions where hydatid disease is endemic and serological results are pending or available, CE should be considered since detection of CL cyst type is identical to the detection of a hydatid cyst not typical for echinococcosis [33].

The CL cases normally present as unilocular anechoic, therefore lacking internal echoes and septa, and these patients are recommended not to be put on treatment until follow-up towards definitive diagnosis is concluded. As such, the seven participants with CL were advised to undertake further investigations in a referral facility, as repeated check-ups would help differentiate a potential CE cyst (CL) from a non-parasitic one. The positive predictive value for serological tests for CE is known to be low [34], so serology was not done. Since this study was time bound, continued follow-up is being done by the county referral hospitals.

Age was unconditionally associated with the presence of simple liver cysts, whereby the odds of having a simple liver cyst increased by 1.09 with every 1-year increase in age (Table 2), equivalent to a 2.37 (1.09^10^) increased odds of having simple liver cysts with every 10-year increase in age. Hydatid disease is a chronic infection; it takes time to develop in humans and may persist in body organs for many years [9]. It is therefore not surprising that the odds of infection in a CE prevalent area would increase with age, and this should be kept in mind when developing future targeted surveillance strategies.

Over the years, western Kenya has had a high worm burden, particularly of soil transmitted helminths and schistosomiasis among preschool [35] and school going children. The Kenyan Ministry of Health, with the support of the WHO, has been implementing a school-based deworming programme using albendazole every three months, and recent studies in Kenya indicate a continuous decline of the incidence of *A*. *lumbricoides*, hookworms, and *T*. *trichuria* among children in Kenyan public schools [36–39]. While continuous uptake of anti-helminthic drugs in the region may have suppressed the incidence of parasitic diseases, there is currently very low evidence that regular anthelmintic treatment would have an effect on CE. The movement of animals from Turkana and Pokot to western Kenya results in an ongoing risk for creation of a new CE econiche. The concern as to whether or not competent parasitic reservoirs have been established to advance the lifecycle is therefore fragile but viable. Our earlier investigations on the ecology of domestic dogs in this region have confirmed that domestic dog scavenging behaviour is associated with proximity to abattoirs dealing in meat from at risk regions [40], such that all elements are in place for the parasite to become established. It is therefore possible that minimal interventions like continuous deworming of dogs, livestock and humans may be interrupting or delaying the establishment of a new disease focus, and this should be investigated further.

Incidental findings of other US-detectable conditions was expected, and our study therefore implemented an information and referral protocol for these cases. Such findings create a challenge because the underlying causes may have significant health implications, and be of great concern to patients [41]. Participants had consented for disclosure and referral of possible incidental findings. Arrangements were in place to hand over any confirmed CE case(s) to the Cystic Echinococcosis in sub-Saharan Africa Research initiative (CESSARi) for follow-up and treatment in collaboration with county hospitals. For other lesions, we committed to providing counselling through the clinician and a referral to a county hospital for follow-up. We therefore did not provide any compensation since we could not follow up on their undertakings.

Incidental abdominal findings included mild to moderate splenomegaly, uterine fibroids, ovarian cysts, polycystic kidneys, and benign prostatic hyperplasia (BPH). Western Kenya is considered endemic for malaria [42], and splenomegaly might be linked to the high incidence of malaria and *Schistosoma mansoni* [43, 44], or malaria and invasive bacteria co-infections [45], in the study area.

In the multivariable logistic regression model, income was statistically associated with abnormal US findings, whereby the odds of having abnormal US findings decreased as the monthly income increased. This corroborates earlier findings in the same region which reported an inverse relationship between economic power and risk of infection by an array of pathogens [46]. In this study, a considerable proportion of the study population lived below the international poverty margin as their earnings were less than US$ 1.90 [47], and would therefore not afford to sustain a treatment process, including surgery, hospitalization and chemotherapy, required for a CE case. Indeed, minimum treatment costs for a case of human CE in a Kenyan government facility is between US$ 600 [48] and US$ 1000 (Zeyhle, personal communication), depending on the severity of the case and cyst location. A single case of CE in a population without insured healthcare may compromise the already meagre resources of the household. Cystic echinococcosis remains a neglected tropical disease [49] mostly affecting the world’s poorest; efforts to control CE [50] must therefore be accompanied by concomitant efforts to improve their standard of living.

Gender was marginally associated with abnormal US findings, whereby males tended to have lower odds of having abnormal US findings, compared to females. However, several of the abnormal findings were naturally “female- related”, such as uterine fibroids and ovarian cysts, which are majorly linked to increased production of oestrogen [51], and female endocrine disorders, respectively. Positive abdominal findings also increased with an increase in age, and among the elderly participants there were reports of BPH (in men) as well as simple liver cysts.

The portable US machine was a valuable resource in such a poor resource setting region. Nonetheless, power outages and power generator breakdowns often delayed the completion of scheduled activities, highlighting the challenges of working in such field conditions. Use of US has not been fully considered a field deployable tool or a point of care option, both due to limited supply and unavailability of expertise. However, a point-of-care tablet-based ultrasound system has been used successfully to perform abdominal ultrasounds in a separate field investigation in western Kenya [52], further illustrating the potential of such tools. The American College of Gastroenterology proposes observation with expectant management for simple liver cysts [53] thus may require use of extra US diagnostic tools to aid management of CLs.

This was a short study designed as an initial assessment of the potential establishment of a new CE focus in a potentially at risk population living near abattoirs where many *Echinococcus granulosus* cysts in livestock have been identified [54] and roaming dogs are abundant [40]. We feel that there are grounds for a larger study to be undertaken in this population, that would potentially involve the routine screening of the population attending health units. We also encourage the consideration of US by health services as a field deployable diagnostic tool to aid epidemiological investigations, leading to early detection of a range of conditions with high disease burden.
